# In-silico and in-vitro study reveals ziprasidone as a potential aromatase inhibitor against breast carcinoma

**DOI:** 10.1038/s41598-023-43789-1

**Published:** 2023-10-02

**Authors:** Ankita Sahu, Shaban Ahmad, Khalid Imtiyaz, Ajeeshkumar Kizhakkeppurath Kumaran, Mojahidul Islam, Khalid Raza, Murugesh Easwaran, Asha Kurukkan Kunnath, Moshahid A. Rizvi, Saurabh Verma

**Affiliations:** 1 Tumour Biology Lab, ICMR-National Institute of Pathology, New Delhi, 110029 India; 2 Department of Computer Science, Jamia Millia Islamia, New Delhi, 110025 India; 3 Department of Bioscience, Jamia Millia Islamia, New Delhi, 110025 India; 4 Molecular and Cellular Medicine, Institute of Liver and Biliary Sciences, New Delhi, 110070 India; 5 Nutritional Improvement of Crops, Plant Molecular Biology Division, International Centre for Genetic Engineering and Biotechnology, New Delhi, 110067 India; 6 Mumbai Research Center, ICAR-Central Institute of Fisheries Technology, Navi Mumbai, 400703 India

**Keywords:** Biological techniques, Cancer, Computational biology and bioinformatics

## Abstract

Aromatase enzyme plays a fundamental role in the development of estrogen receptors, and due to this functionality, the enzyme has gained significant attention as a therapeutic for reproductive disorders and cancer diseases. The currently employed aromatase inhibitors have severe side effects whereas our novel aromatase inhibitor is more selective and less toxic, therefore has greater potential to be developed as a drug. The research framework of this study is to identify a potent inhibitor for the aromatase target by profiling molecular descriptors of the ligand and to find a functional pocket in the target by docking and MD simulations. For assessing cellular and metabolic activities as indicators of cell viability and cytotoxicity, in-vitro studies were performed by using the colorimetric MTT assay. Aromatase activities were determined by a fluorometric method. Cell morphology was assessed by phase-contrast light microscopy. Flow cytometry and Annexin V-FITC/PI staining assay determined cell cycle distribution and apoptosis. This study reports that CHEMBL708 (Ziprasidone) is the most promising compound that showed excellent aromatase inhibitory activity. By using better drug design methods and experimental studies, our study identified a novel compound that could be effective as a high-potential drug candidate against aromatase enzyme. We conclude that the compound ziprasidone effectively blocks the cell cycle at the G1-S phase and induces cancer cell death. Further, in-vivo studies are vital for developing ziprasidone as an anticancer agent. Lastly, our research outcomes based on the results of the in-silico experiments may pave the way for identifying effective drug candidates for therapeutic use in breast cancer.

## Introduction

Breast cancer (BC) is the most prevalent cancer worldwide and the leading cause of cancer death among women, reported primarily in females (> 99%) and very rarely in males (< 1%). The development and progression of breast carcinoma depend on genetic abnormality and hormonal deregulation^1^. The worldwide incidence of BC is 25% of all cancers in women. It disproportionately affects individuals in low- and middle-income countries where it is women’s most common cause of death^2^. The incidence of breast cancer is most common after menopause (World Cancer Research Federation WCRF (https://www.wcrf.org/dietandcancer/breast-cancer). Numerous pathophysiological reasons like gene mutations (especially breast cancer genes BRAC1 and BRAC2), inherited genetic predisposition, exposure to hormones (estrogen and progesterone), diet-related, and exposure to harmful carcinogenic chemicals in the environment and unhealthy lifestyles lead to breast cancer development^3–5^. Several potential targets, such as vascular endothelial growth factor, epidermal growth factor receptor, and numerous enzymes, were reported to be identified in cancers and diseases associated with the reproductive system^6–10^.

Aromatase, also known as Cytochrome P45019A1, is a membrane-bound microsomal enzymatic complex that is a heme protein, present in the endoplasmic reticulum of estrogen-producing cells. It comprises of a prosthetic heme group and a polypeptide chain of 503 residues^11,12^. It consists of 9 exons and a 5′-untranslated region on the human CYP19 gene (localization of 15q21.1 region), stretching ~ 123 kb. The aromatase enzyme catalyzes the conversion of androgen precursors to aromatic estrogens^13,14^, and thus is a promising target that could be used to address reproductive disorders and malignancies^15^. The transformation occurs in the androgen-specific cleft containing hydrophobic and polar residues that stimulate cellular proliferation in breast cancer^13,16^. The aromatase enzyme oxidizes and subsequently removes the methyl group at the A ring to bring it into an aromatic state, thus converting androgen into estrogen^13^. Estrogen surge in the breast tissues is the primary hormonal requirement for the progression of tumorigenesis. Aromatase is a rate-limiting enzyme found in several human tissues, subcutaneous fat, placental syncytiotrophoblasts, ovarian granulosa cells, skin fibroblasts, adipose tissue, osteoblasts of bone, brain and cancerous as well as normal breast tissues^15,17,18^. The source of residual estrogen is solely non-glandular especially subcutaneous fat. The estradiol level in breast carcinoma tissues is several times higher than in the blood because of its overexpression in such tissues^4,19^. This increases the significance of monomeric aromatase enzyme inhibition as a front-line therapeutic intervention in estrogen hormone-dependent breast cancer. Only influential post-menopausal women are most frequently used to inhibit tumour growth and breast cancer cell proliferation^16,20^.

A drug discovery process originates when research is undertaken to find suitable drugs that can cure clinical conditions. The initial step of the research process starts with bioinformatics analysis with the identification and validation of biological targets^21^ that include biomolecules like protein, gene and RNA, which can be quantified by experimental methods using in-vitro and in-vivo models. The protein’s functioning can be studied at the atomic level using different techniques and algorithms for molecular docking, ADMET profiling and simulation of their three-dimensional structures. The screening of novel compounds capable of inhibition of aromatase enzyme is essential for biomedical designing of drugs. In this study, ChEMBL database was the vital source from which compound was screened for drug discovery in the biological system. Biocomputational tools are the key technology for computational biology and health informatics to develop lead compounds^22^. Selecting appropriate protein structures from searchable drug databases requires molecular docking strategies to find desirable biological and chemical features. It is widely known that in-silico-based docking studies, residue-protein interaction patterns, ADMET properties, and MD simulations help identify highly appropriate drugs/molecules which cuts time and cost, and prevents adverse consequences of preclinical studies^23^. Thus, high-performing computational algorithms are required for the drug designing process. Molecular docking and MD simulation strategies were used in our study to identify a potent inhibitor of the aromatase target.

**Figure 1:**
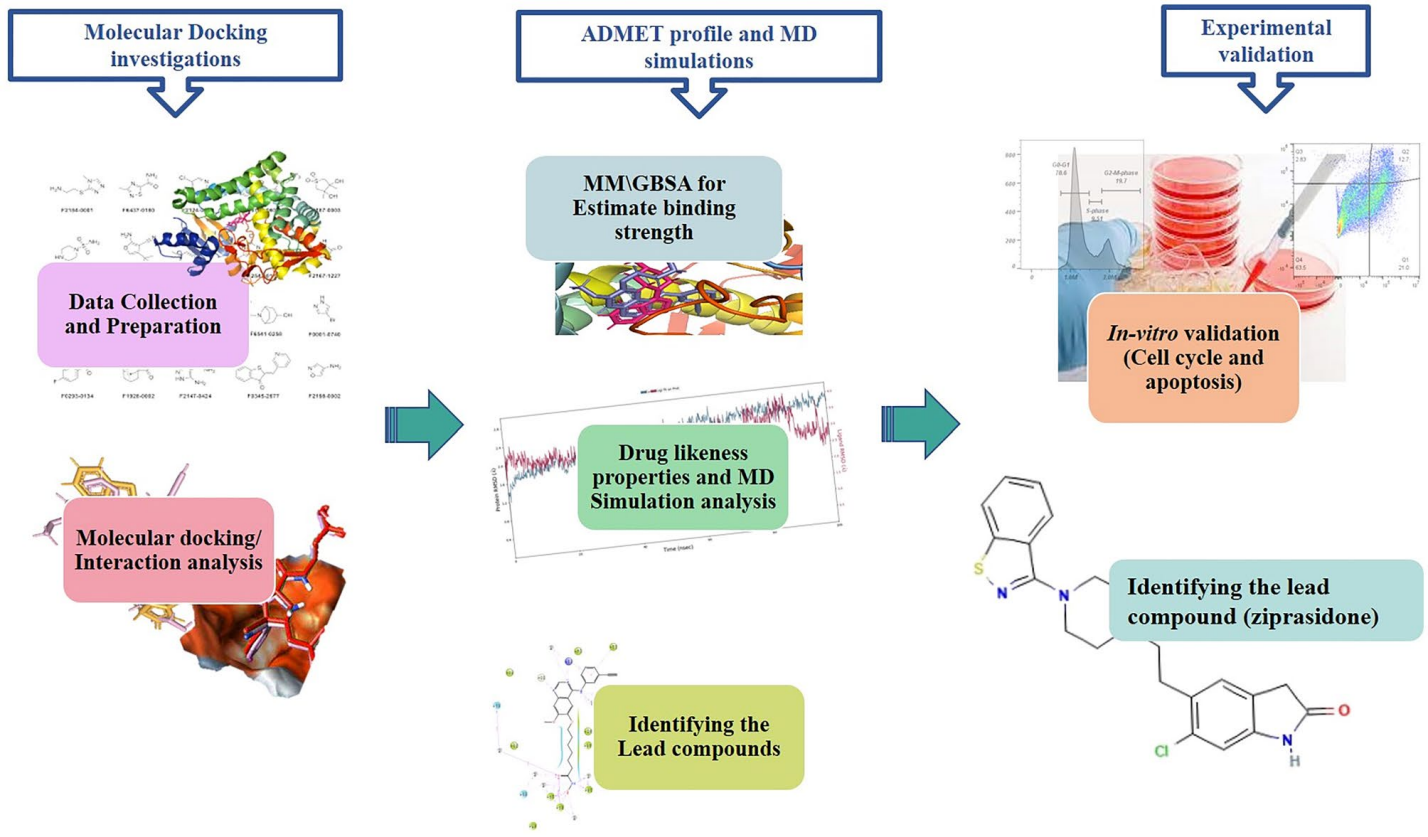
A complete computer-aided drug discovery and in-vitro approaches.

This study aims to identify aromatase inhibitor using an in-silico and in-vitro approach. Briefly, the study is outlined as follows: ChEMBL database screening with the aromatase target protein and exploration of the various in-silico strategies for regulating the biological processes involved in breast cancer progression and validation of the compound in *in-vitro* conditions. Figure 1 illustrates the in-silico and in-vitro framework.

## Results

### In-silico studies

Computational studies such as molecular docking, ADMET profiling and MM/GBSA calculations have provided satisfactory results. The complex was then subjected to MD simulation for 100ns in water medium, and the resultant deviations, fluctuations and simulation interaction diagrams were analyzed. Further, the detailed results of the analysis are as follows.

### 3D-structure modelling and validation

**Figure 2:**
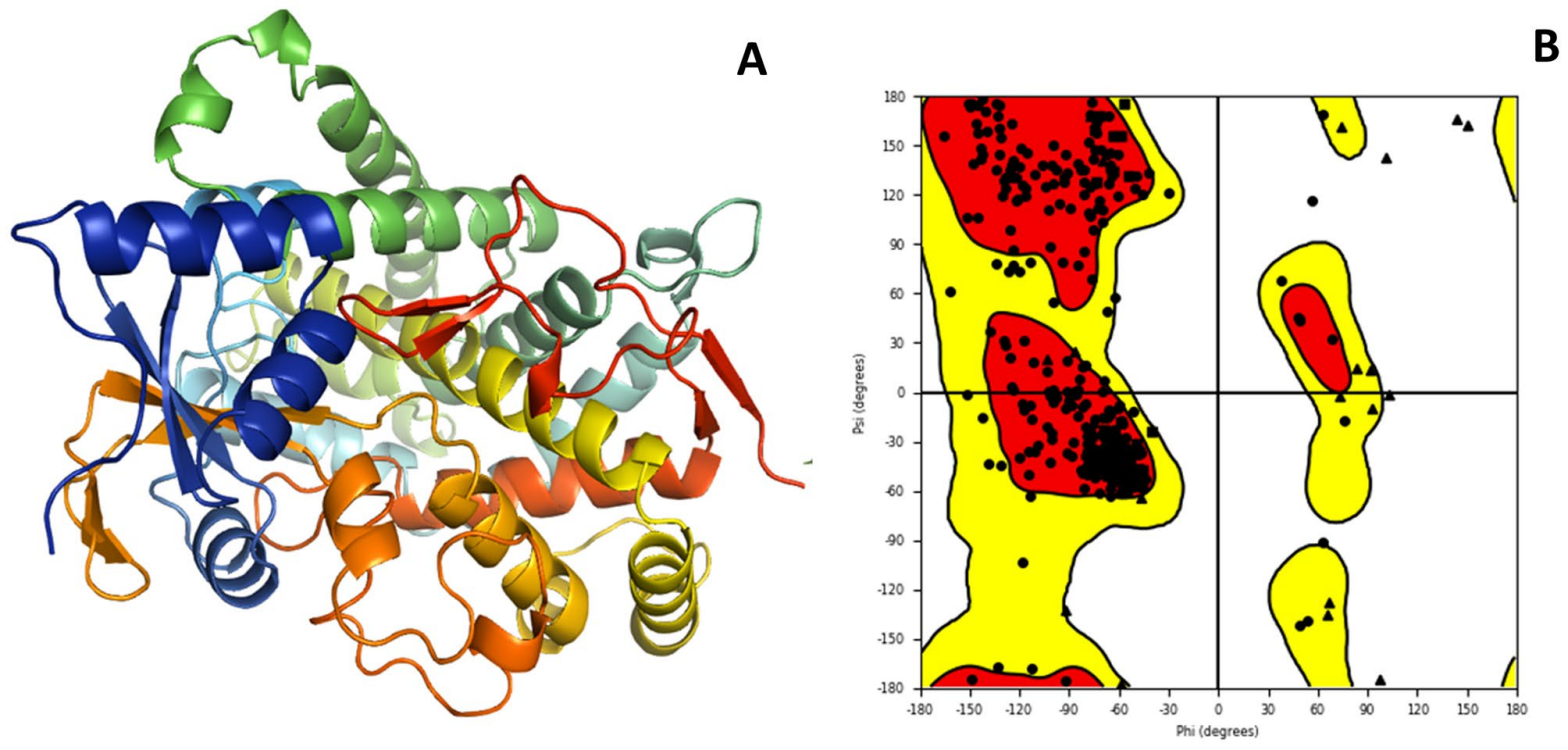
(**A)** 3D Crystal structure of aromatase (PDB ID:3EQM), and (**B**) Ramachandran plot of prepared structure.

Ramachandran plot for the selected aromatase targeted protein was downloaded from protein data bank (PDB ID 3EQM) represented in Fig. 2A, assessing the stereochemical quality of the protein structure. PROCHECK server checks the stereochemical properties of predicted model that generates the Ramachandran plot, as depicted in Fig. 2B. Residues in the beta-conformation are negative, followed by 0 to − 60 psi angles (ψ) and 0 to − 90 in the phi angles (ϕ) are positive, showing dense conformation of residues in the targeted aromatase protein. Based on the results, 94.33% of residues were found in the most favoured region, 4.67% amino acid residues were likely found in the additional allowed region, 1% in the generously disallowed area and none of the residue (white region) in the disallowed region.

### Binding affinity calculation

The VSW has produced 17 top performing ligands after computations of HTVS, SP and XP sampling algorithms and the top performing ligand was ziprasidone which are shown in the ST1 (Supplementary Table 1). The bound structure of receptors with ligands is considered a therapeutic target for breast cancer treatment and we have docked the native ligand as well, to check how our proposed compound advances and found that ziprasidone has better binding affinities which are shown in the ST2 (Supplementary Table 2) and SF1 (Supplementary Fig. 1). The docking calculation details the binding energies between the selected drugs and the carrier systems. The Glide Score was analyzed, and the top-ranked compound was found through docking results. The attractive force and the binding affinity of the interacting protein–ligand docked structure determines the binding affinity. The binding affinity values for the docked structure of aromatase protein are followed by Table 1.

**Table 1:** Showing the docking score and other energy scores generated during the interaction calculations.

PDB	Docking score	MM\GBSA	Prime Hbond	Prime vdW	mol MW	Ligand efficiency sa	Ligand efficiency In
3EQM	− 9.837	− 88.31	− 200.37	− 2547.18	412.936	− 1.067	− 2.271

Docking and MM\GBSA are in Kcal/mol.

**Figure 3:**
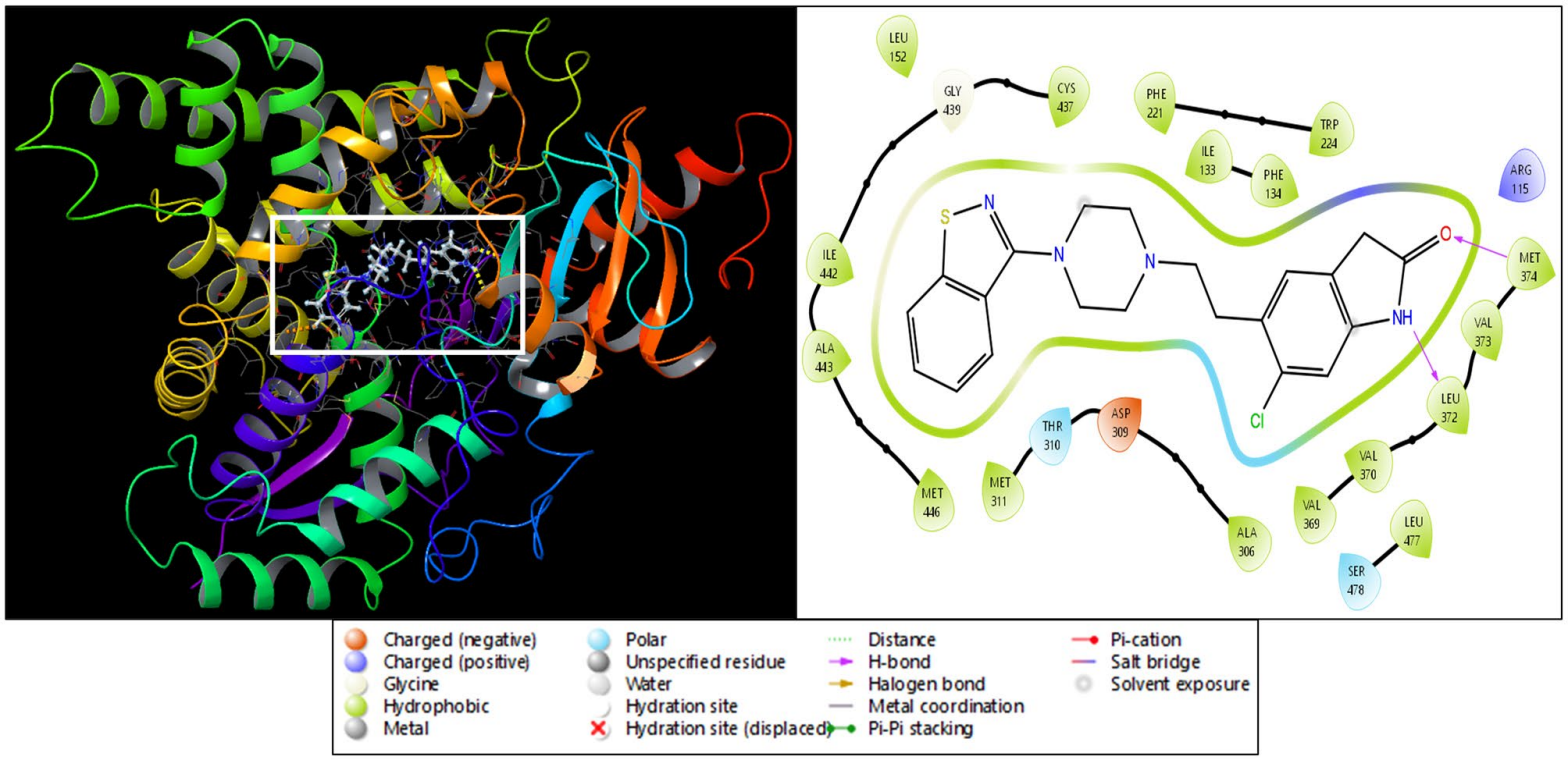
Ligand interaction diagram of the 3EQM and Ziprasidone where VAL369 and VAL368 interact with the O atom and the same VAL370 interacts with the OH atom whereas the PRO429 interacts with the NH atom of the ligand.

The lowest glide score (docking score) characterizes a more agreeable binding and in our case LEU322 has formed hydrogen bond with NH atom while MET374 has formed hydrogen bond with ‘O’ atoms of Ziprasidone producing − 9.837 kcal/mol. At the binding site, the binding conformation of the aromatase receptor is recognized for their remarkable inhibitory effect against aromatase activity as shown in Fig. 3. Further, the MM/GBSA calculated for binding free energy score with OPLS-2005 which gives a much more accurate scoring of the ligand pose than the XP score. The scoring was elevated in the ligand ranking high i.e. compound CHEMBL708 which shows good aromatase inhibiting tendency with ΔGMM-GBSA values of − 88.31 kcal/mol.

### Molecular and principal descriptors of the ligand

Several potential therapeutic agents fail during clinical trials due to their unfavourable ADMET properties. ADME calculation aids to determine the predicted drug-likeness. The calculated ADMET endpoints are summarised in (Table 2). The oral availabilities of the compounds help to ascertain the molecular properties implicit to drug-likeness that work with five descriptors, including molecular weight (MW) ≤ 500 Da, QPlogPo/w value ≤ 5, Hydrogen acceptor ≤ 10, Hydrogen donor ≤ 5, Topological Polar Surface Area (TPSA) < 5. These criteria follow the Lipinski’s rule of five^24,25^. Twenty-two parameters describe the pharmacokinetic profile, and most of the values lie within the allowable range. These values are suitable for the selected active ligand and the number of likely metabolic reactions also fall within the permissible range. A detailed analysis of the molecular dynamics simulation of aromatase protein and the docked structures has been carried out for various parameters. The ADMET profile of the compound CHEMBL708, that satisfies the binding affinity and Lipinski’s rule of five (Table 2).

**Table 2:** Showing the QikProp or ADMET result of ziprasidone (CHEMBL708) against the standard values.

Properties or descriptor	QikProp standard values	Ziprasidone	Properties or descriptor	QikProp standard values	Ziprasidone
Type	N/A	Small	QPlogPo/w	− 2.0 – 6.5	3.905
#stars	0–5	0	QPlogS	− 6.5–0.5	− 5.542
#amine	0–1	1	CIQPlogS	− 6.5–0.5	− 5.276
#amidine	0	0	QPlogHERG	concern below − 5	− 6.759
#acid	0–1	0	QPPCaco	< 25 poor, > 500 great	211.765
#amide	0–1	0	QPlogBB	− 3.0–1.2	− 0.146
#rotor	0–15	3	QPPMDCK	< 25 poor, > 500 great	382.649
#rtvFG	0–2	0	QPlogKp	− 8.0 − 1.0	− 4.483
CNS	− 2 (inactive), + 2 (active)	1	IP(eV)	7.9–10.5	8.533
mol MW	130.0–725.0	412.936	EA(eV)	− 0.9–1.7	0.876
Dipole	1.0–12.5	5.155	#metab	1–8	3
SASA	300.0–1000.0	699.911	QPlogKhsa	− 1.5–1.5	0.661
FOSA	0.0–750.0	231.737	HumanOralAbsorption	N/A	3
FISA	7.0–330.0	112.511	% HumanOralAbsorption	> 80% is high, < 25% is poor	91.438
PISA	0.0–450.0	251.018	SAfluorine	0.0–100.0	0
WPSA	0.0–175.0	104.645	SAamideO	0.0–35.0	0
Volume	500.0–2000.0	1236.056	PSA	7.0–200.0	67.356
donorHB	0.0–6.0	1	#NandO	2–15	5
accptHB	2.0–20.0	6	RuleOfFive	maximum is 4	0
dip^2/V	0.0–0.13	0.0214992	RuleOfThree	maximum is 3	0
ACxDN^0.5/SA	0.0–0.05	0.0085725	#ringatoms	N/A	24
glob	0.75–0.95	0.7958192	#in34	N/A	0
QPpolrz	13.0–70.0	43.784	#in56	N/A	24
QPlogPC16	4.0–18.0	13.271	#noncon	N/A	5
QPlogPoct	8.0–35.0	19.882	#nonHatm	N/A	28
QPlogPw	4.0–45.0	10.626	Jm	N/A	0

The logP distribution describes the lipophilicity of a compound that denotes the partition coefficient. The range of hydrogen bond acceptor (H.B.A.) and hydrogen bond donor (HBD) of the compound CHEMBL708 were 1 and 3.5, respectively, which indicates that the compound has a drug-like favourable range. The physiochemical descriptors like SASA, FOSA, FISA, PISA (π component of SASA), QPlogPC16, QPlogPoct, and QPlogPw were also selected for this study, and all parameters were observed to be within the normal range.

The permeability of the gut-blood barrier is predicted by the Caco-2 parameter. It is a non-active transport evaluation used in blood absorption assay and is expressed in nm/s^26^. This parameter helps to identify and evaluate the approximate passage of substances through the gut wall. The Caco-2 parameter for the compound CHEMBL708 was 137.54nm/s. The QPlogBB partition coefficient is used to predict the compound’s brain/blood partition for CNS^27^. The QPlogBB partition coefficient for CHEMBL708 was found to be in the range -0.146 and − 2.294 which shows that the top compound was active with the acceptable range in CNS activity. The QPPMDCK is used to predict blood–brain barrier (BBB) penetration. The optimal value lies in the range 382.649 and 112.052 nm/sec of the selected drugs. QplogKp calculates the permeability of penetrating the drugs/compounds through the skin. The equation projects the maximum trans-dermal transport rates:

$${\text{Jm }} = {\text{ Kp }} \times {\text{ MW }} \times {\text{ S}}.$$

Jm is the trans-dermal transport rate expressed in the unit of μg cm, Kp symbolizes the skin permeability and molecular weight (MW), and S denotes aqueous solubility. QplogKp for the compound CHEMBL708 was − 5.043 (mol dm^–3^).

QPlogKhsa predicts the plasma-protein binding of compounds, which may bind to human serum albumin, glycoprotein, globulins, and lipoprotein and has an inverse correlation to the target obtainability. Drug efficacy is unswervingly influenced by the distribution of the drugs through the bloodstream, their binding ability and the accessibility of drugs to their target. Consequently, a lower degree of binding to plasma proteins is essential for drugs to be effective. Compound CHEMBL708 has QPlogKhsa value of 0.12 nm/s that is optimum. QPlogHERG is an essential parameter for predicting the blockage of human ether-a-go-go-related gene (hERG) potassium channel in the cardiac and nervous system to predict the cardiac toxicity of drug molecules^28,29^. HERG K + channels have QPlogKhsa > − 5. The channel also has a modulating function in the nervous system. The ADME investigation of CHEMBL708 displayed that all parameters except CIQPlogS (score 6.6) and QPlogHERG (score 5.9) showed favourable values for drug-likeness, metabolism, pharmacokinetics and criteria.

### Molecular dynamics simulation of protein–ligand complex

The simulation of the docked structure was performed after the equilibration phase for 100 ns and several metrics were plotted to prove the stability of the structure. We have extensively analyzed the trajectories and presented the RMSD, RMSF and intermolecular interactions during the simulation period. Further, the detailed analysis is as follows-

#### Root mean square deviation and root mean square fluctuations

**Figure 4:**
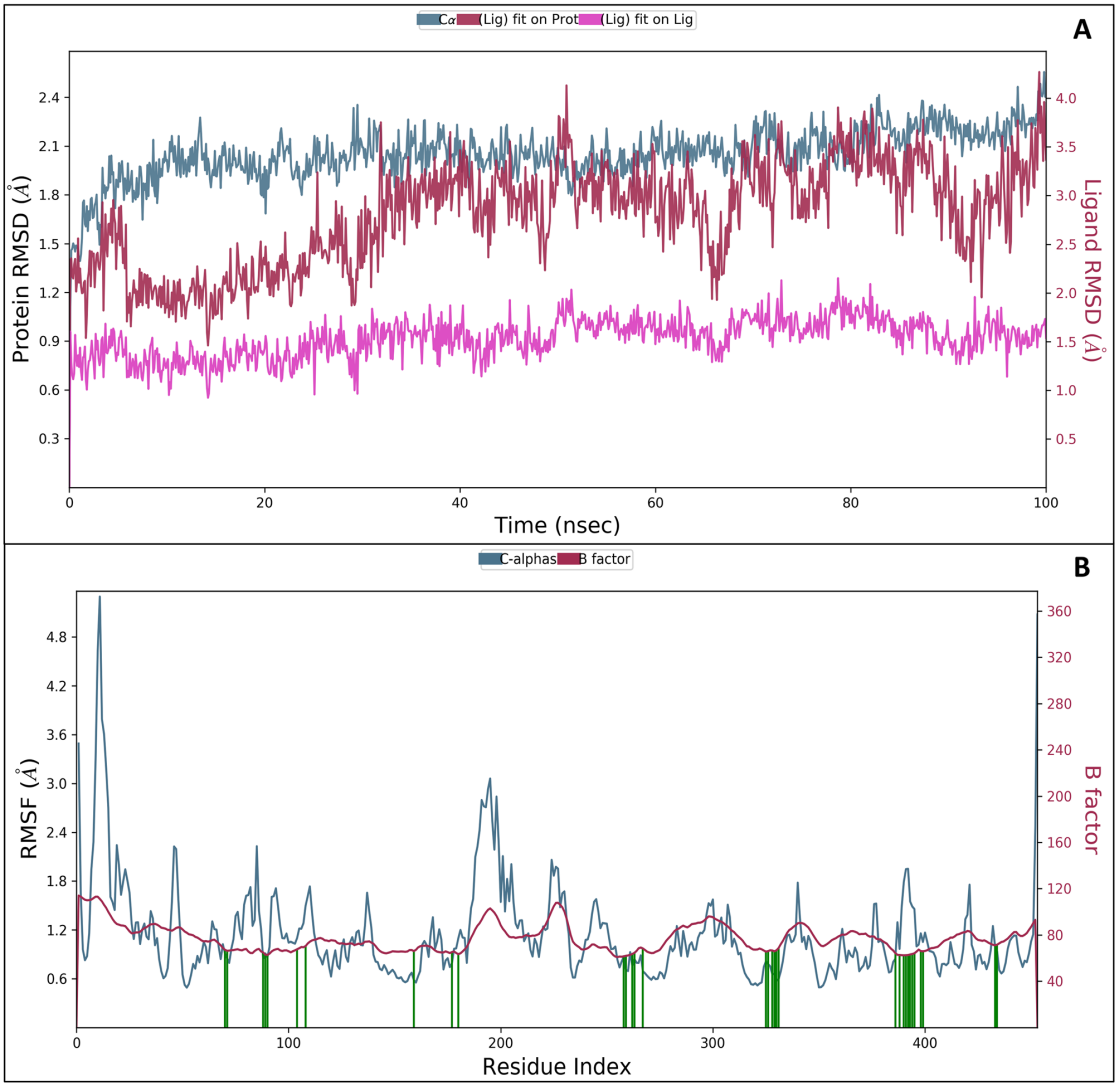
MD. Simulation results in (**A**) Root Mean Square Deviation (RMSD) and (**B**) Root Mean Square Fluctuations (RMSF) where the blue colour represents the Cα fluctuations while the green line represents the Ziprasidone contacts with residues.

The root means square deviation (RMSD) is used to analyze the exact deviations during the simulative period, and in our analysis, we have extensively analyzed the complete trajectories of the deviations. Figure 4A illustrates the RMSD for Ziprasidone CHEMBL708 and the targeted protein, from zero to hundred nanoseconds. At 0.10 ns, the protein showed an initial deviation of 1.11 Å while the ligand showed a deviation of 2.33Å. This initial deviation is a result of a change in the solute medium, added Cl^-^ ions, and the induced heat. After the initial deviations that can be ignored, the protein and ligand both showed least deviation of 2.56Å and 3.96Å at 100 ns. After ignoring the initial deviation of 1.11Å, a cumulative deviation of 1.45Å was observed for protein while for the Ziprasidone it was 1.63 Å which is less than 2Å, meaning the protein and ligand structures were stable. The deviations in the mentioned duration can be because of interactions or addition of ions and sudden heat in the system. Moreover, the ligand followed the same trajectory again and showed stable performance. Overall, the protein and ligand deviations were less than 2Å indicating that the protein–ligand complexes were stable. In Fig. 4A we have shown the deviation of Cα in blue, ligand fit of protein in red and ligand fit on ligand in pink to make it clearer.

For the Root Mean Square Fluctuations (RMSF), we analyzed the Cα along with the ligand contacts to know which amino acid residues are in contact with the ligands during the MD simulation (Fig. 4B). SER114, ARG115, ILE132, ILE133, PHE134, PHE148, LEU152, PHE203, PHE221, TRP224, GLU302, MET303, ALA306, ALA307, MET311, VAL369, VAL370, LEU372, VAL373, MET374, ARG375, PHE430, PHE432, PRO434, ARG435, GLY436, CYS437, ALA438, GLY439, ILE442, ALA443, LEU477 and SER478 are among the residues which interacted with the ligand during the simulation period (Fig. 4B). A few residues that went beyond 2Å of fluctuation are SER45, TYR52-LEU59, GLY63, SER90, GLY91, GLU129-LYS243, GLU245, LYS249, THR268, and ASN496. The RMSF analysis concluded that only a few residues went beyond 2 Å fluctuation, which is typical as amino acid residues try to interact with the ligands during the simulation period.

#### Simulations interaction analysis

**Figure 5:**
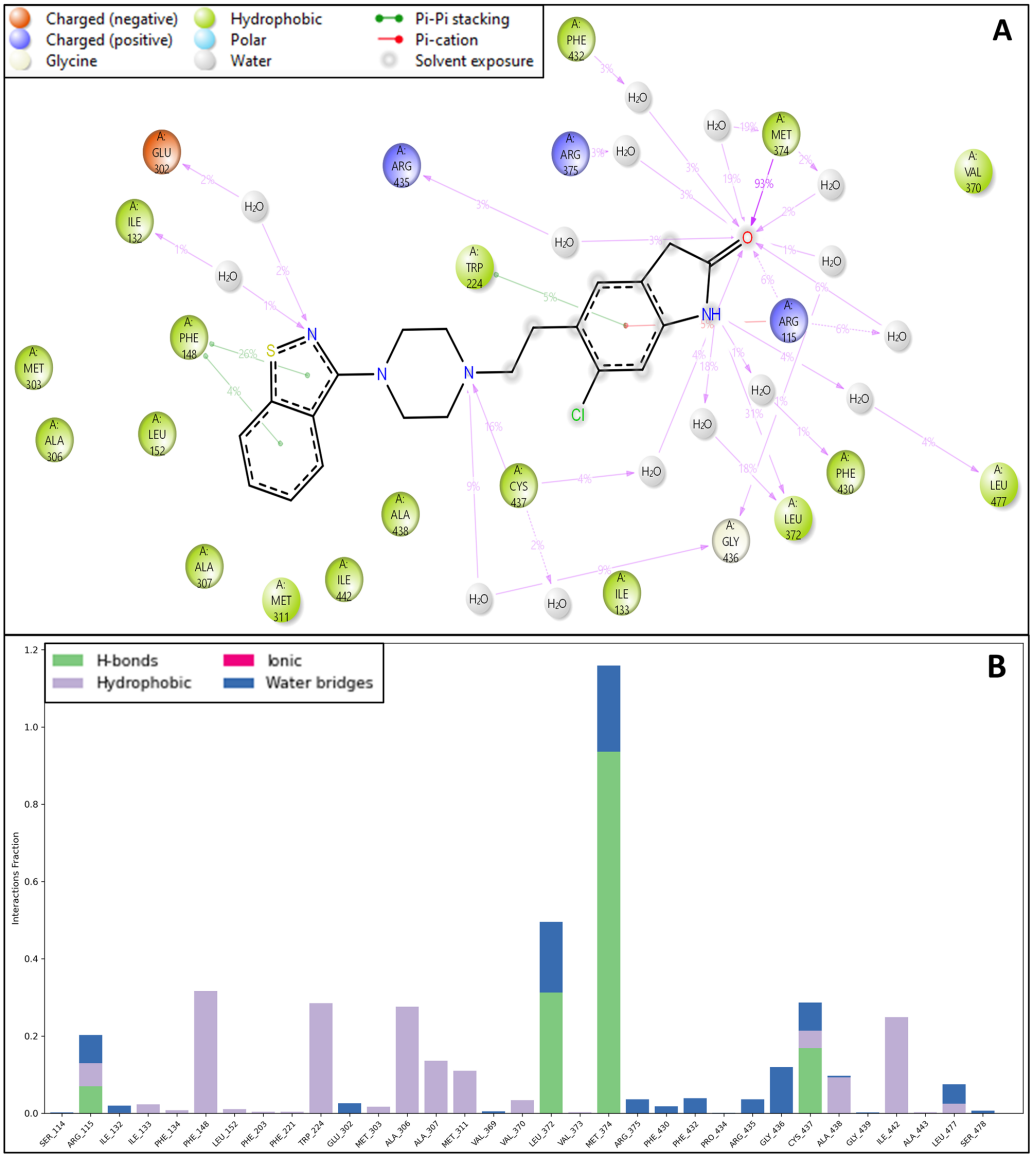
Simulation interaction diagram of Ziprasidone (**A**) 2-D interactions representation of the protein–ligand and (**B**) histogram representation of the count of interaction during the simulation period.

Strong interactions between the ligand and the targeted protein aromatase are essential in strengthening the receptor-ligand stability, such as hydrogen bonding, hydrophobic interactions, pi cation, and salt bridges, which were visualized while analyzing the simulation interaction diagrams. 2D interaction maps of ChEMBL-based docked complex portraying the preservation of interactions through the simulation trajectory are shown in the interaction diagram. The acceptor hydrogen bond (red) and donor hydrogen bond (yellow) profiles of co- crystal ligand were close to compound CHEMBL708. The counts of acceptor and donor bonds remarkably emphasized the significant interactions of hydrogen bonds. 2D interaction maps depicted the hydrogen bond, pi-pi, and pi-cation interaction of the compound CHEMBL708 with the backbone amino acid residues ARG435, PHE432, ARG375, MET374, ARG115, LEU477, PHE430, LEU372, GLY436, TRP224, CYS437, PHE148, ILE132, and GLU302 (Fig. 5A).

These interactions show the involvement of high-energy *aromatic amino acid* residues in packing the adenine ring in the targeted protein. Ionic interactions (side chain metal mediate) were also observed in the amino acid residues. Further, Fig. 5B describes the count of the different intermolecular interactions, such as hydrogen bonds, hydrophobic interactions, and water bridges assembled by each pocket residue with the ligand binding site. Compound CHEMBL708 was efficiently docked and validated for better-quality docking results. Some residues displayed a similar hydrogen bonding and hydrophobic interaction with the amino acid residues.

### In-vitro study

#### Antiproliferative activity in cancer cells

**Figure 6:**
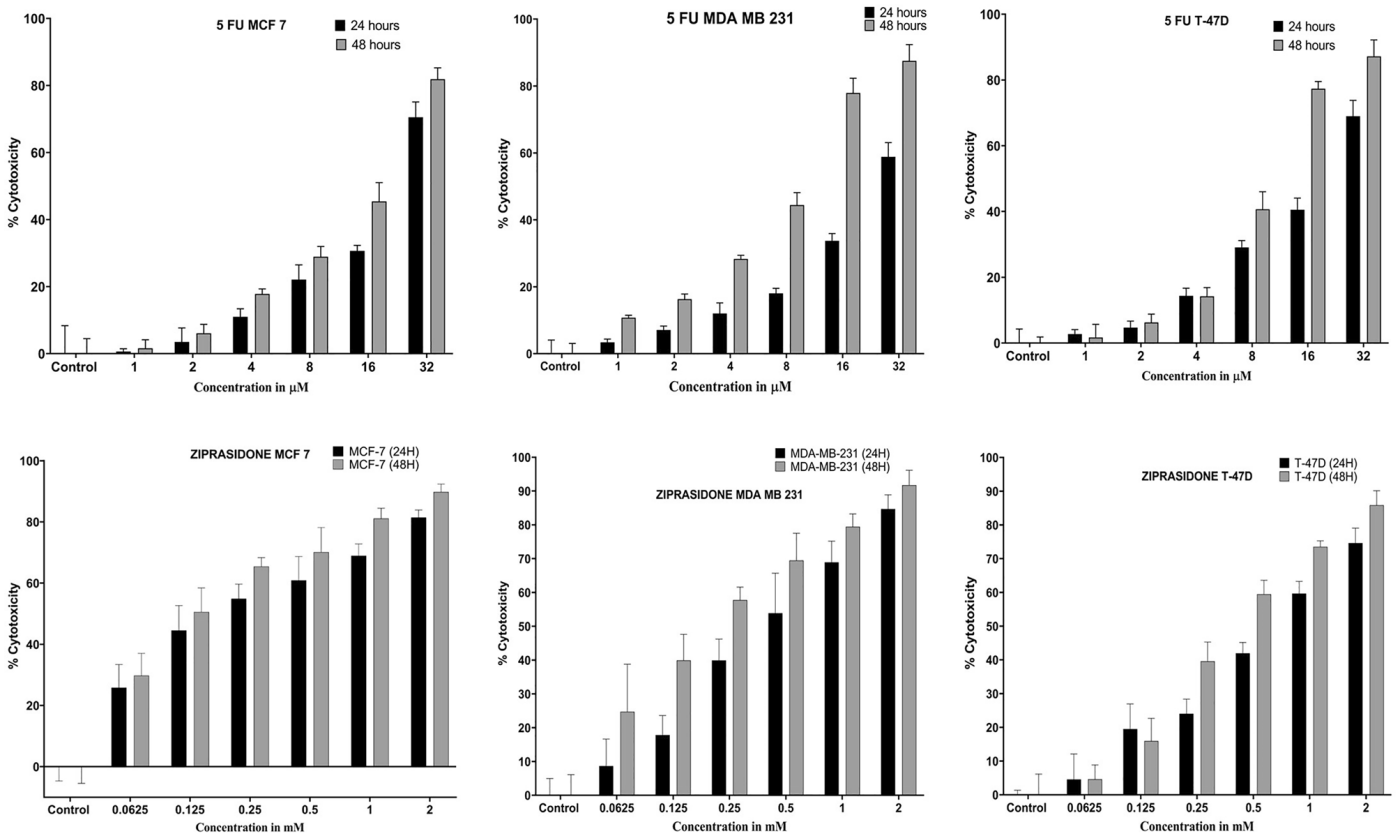
Antiproliferative activity of control (5FU) and ziprasidone in MCF-7, MDA MB 231 and T47D cells at 24 and 48 h post-treatment.

The anticancer effect of ziprasidone on the growth of MCF-7, MDA-MB-231 and T47D was determined by the MTT assay, as per method described earlier^30^. In the present experiment, cells were treated with 5-FU as control, parent compound and these compounds at 2, 1, 0.5, 0.25, 0.125, and 0.0625 mM for 24 h and 48 h. The dose–response curve was used to calculate the IC_50_ value- the drug concentration required to reduce cell proliferation by 50% against an untreated control. The IC_50_ values for Ziprasidone in MCF-7 cells were found to be 0.260 mM and 0.158 mM at 24 h and 48 h respectively. For MDA-MB-231 cell lines, the IC_50_ values were 0.532 mM and 0.27 mM at 24 h and 48 h, respectively (Fig. 6). Next, the IC50 values in the case of the T47D cell line were 0.608 mM at 24 h and 0.336 mM and 48 h. The results indicate that there is a significant antiproliferative effect compared with control that is dose-dependent as seen in Fig. 6.

#### Effects of ziprasidone on cell morphology

**Figure 7:**
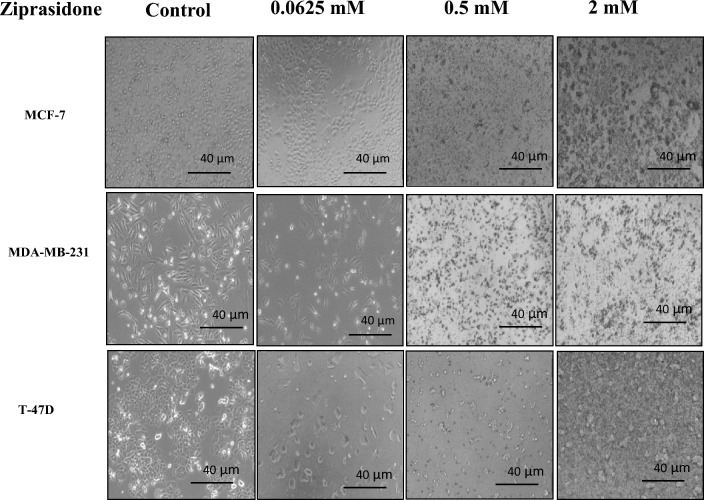
Effect of Ziprasidone on cell morphology of MCF-7, MDA MB 231 and T47D cell lines at 24 h post-treatment. Scale of images are displayed on the on the figure at magnification of 20 × tenfold under inverted microscope.

Morphological changes were recorded using a microscope in MCF-7, MDA-MB-231 and T47D cells. The fluoropyrimidine, 5-fluorouracil (5-FU) is a cytotoxic chemotherapy medication and antimetabolite drug used as a standard positive control drug commonly used to treat cancer. For all treated cells, the images were observed at 24h, and the images were captured using a phase-contrast light microscope. While in the control group, cell shape was not changed, in Ziprasidone treated groups, there was a substantial change in cell morphology, and cell debris of dead cells was also seen. The treatment with Ziprasidone in MCF 7, MDA MB and T47D cells at different concentrations at 24h resulted in round shape and size reduction (Fig. 7).

#### Cell cycle analysis

**Figure 8:**
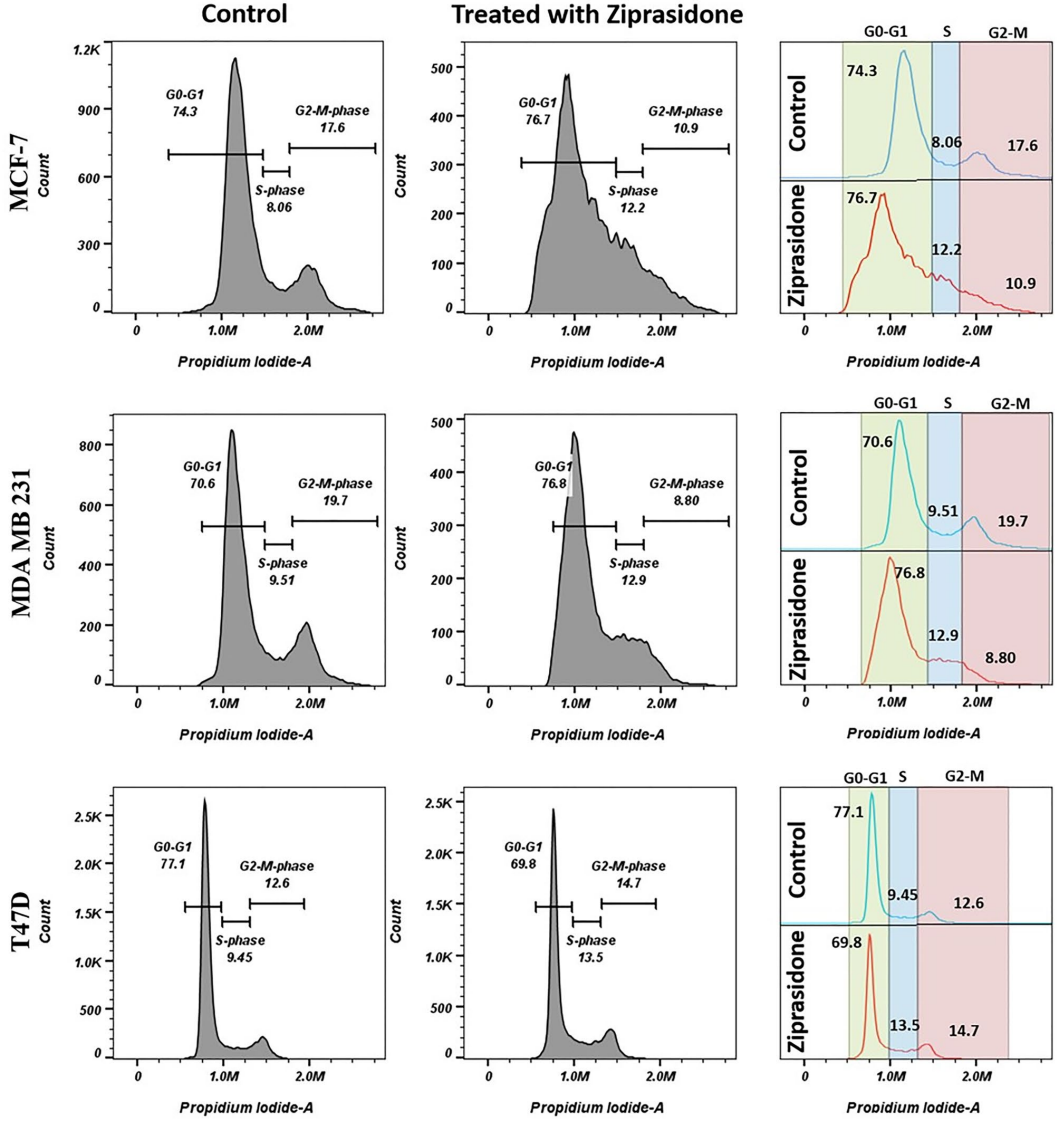
Cell cycle analysis in control and different cell lines (MCF-7, M.D.A. MB 231 and T47D) treated with drugs.

Selected compounds exert growth-inhibitory effects on different cell lines by arresting the cell cycle at specific phases. The in-vitro screening results in Fig. 8 show that ziprasidone significantly increased the cell count in the S phase from 8.06 to 12.2% in MCF-7 cell line from 9.51 to 12.9% in MDA-MB-231 cell line and from 9.45 to 13.5% in T47D cell line in comparison with the control. The statistical significance between individual group of data was recorded at p < 0.05 and compared with control.

#### Annexin V binding assay

**Figure 9:**
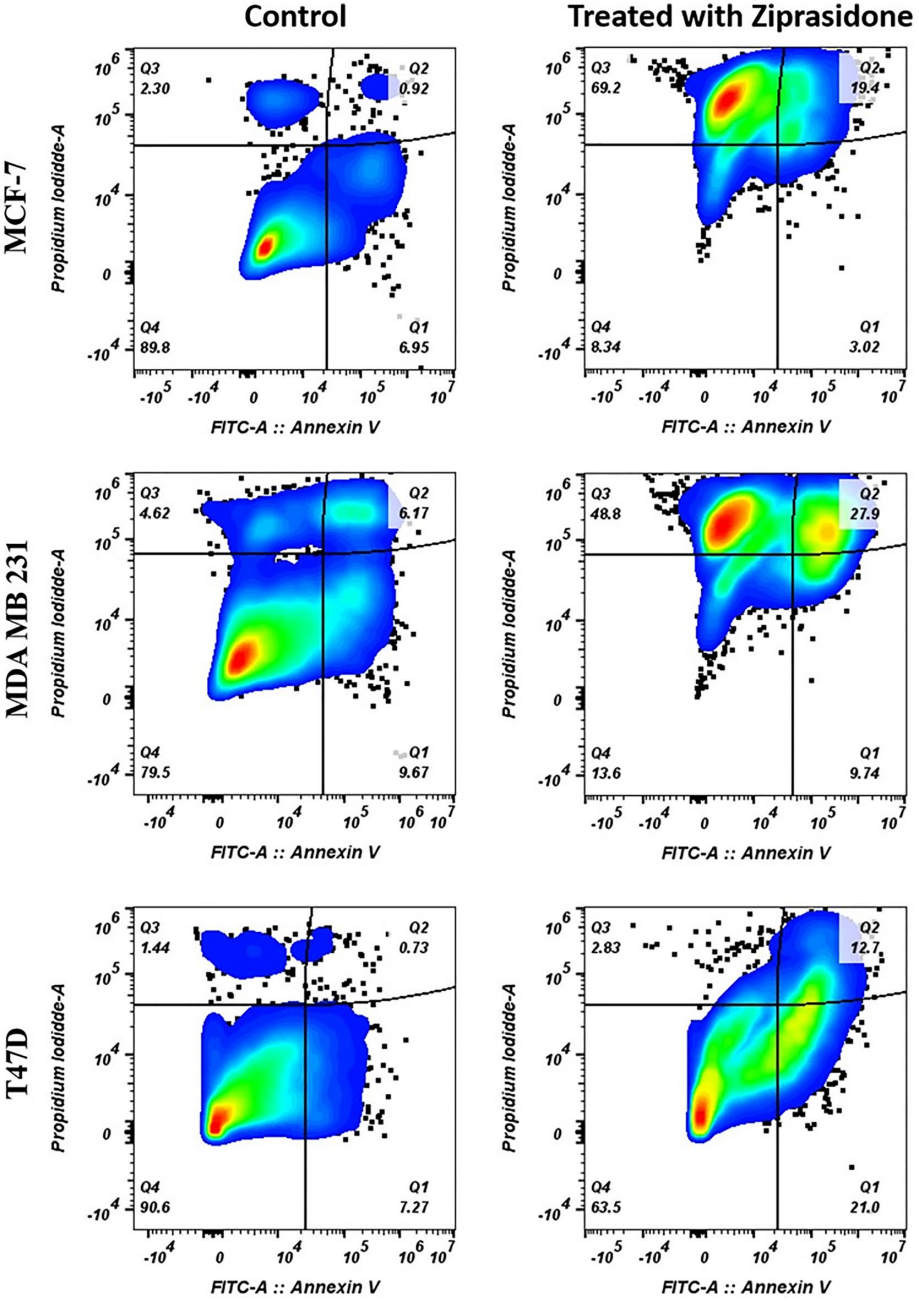
The proapoptotic effect of ziprasidone on MCF-7, MDA-MB 231 and T47D cell lines after 48 h.

Ziprasidone was further investigated to evaluate its effect on apoptosis. It is a pathway leading to programmed cell death. To analyze the effect of ziprasidone on apoptosis in different cell lines, we applied FITC-Annexin V and PI double staining for a flow cytometry assay. MCF-7 cells were treated for 24h with different concentrations of ziprasidone and analyzed by flow cytometry^31^. Figure 9 shows an increased cell death ratio between early and late apoptosis with increasing ziprasidone concentration. Remarkably, 2.77% of the cell population underwent the necrotic phase (Q1 quadrant; Fig. 9) at all concentrations of ziprasidone treatment. When comparing the early and late apoptosis and the necrosis phase, the number of cells was significantly high in the necrotic phase than in the early phase.

#### Aromatase activity assay

**Figure 10:**
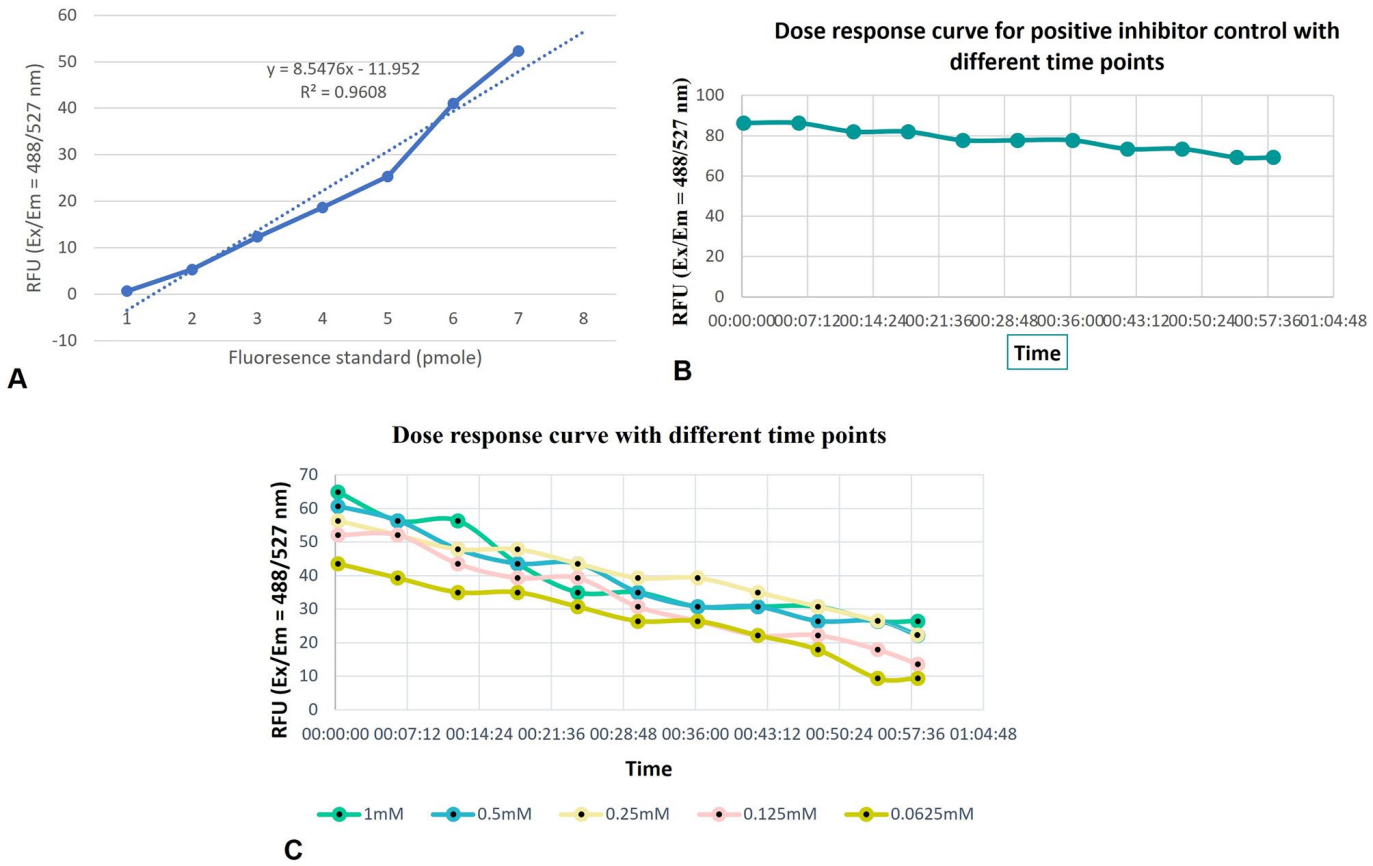
(**A**) Standard curve of Aromatase Substrate metabolite fluorescence. (**B**) Reaction kinetics of recombinant human aromatase enzyme positive inhibitory control (letrazole) at 37 °C. (**C**) Dose–response curves for ziprasidone compound with different concentration.

Next the effect of aromatase inhibitor on ziprasidone was investigated. The proposed mechanism showed effectiveness of detection strategy for detection of enzymatic activity aromatase and ziprasidone. The results of the aromatase activity for the ziprasidone are summarised in Fig. 10A–C. For comparison, the positive control was set and the sample of ziprasidone was calculated accordingly. The measurements were recorded within the linear range of the reaction with the reaction temperature and experimental conditions. In the form of duplication, the different concentration of test sample and positive control were performed. Aromatase fluorometric enzymatic assay were done to screen for the enzyme specific inhibition. To evaluate the effectiveness of the test compounds against aromatase enzyme, a minimum dose that causes 50% inhibition (IC_50_) was determined using serial concentrations (1, 0.5, 0.25, 0.125 and 0.0625 mM) for the positive control samples (letrozole), summarised in Fig. 10B. From the obtained data, (Fig. 10C),

it was observed that the test compound might be of good safety profile as they could inhibit the aromatase enzyme at higher concentrations (IC_50_ = 64.9764 mM) at 1mM test sample. The present outcome was showed the significantly inhibited aromatase with percent activities of 64.9764, 60.7026 and 56.4288% at 1, 0.5 and 0.25mM, respectively, while the lowest concentration showed weaker inhibitory effects.

## Discussion

This study used in-silico and in-vitro approaches to investigate the anticancer effects of the particular compound ziprasidone. The molecular docking approach is considered an emerging field for rational drug design and development and, therefore, is gaining significance in biomedical science^28^. Here, computer-aided approaches were adopted to predict whether or not the selected compound has anticancer potential. Aromatase-targeted protein was selected (PDB ID: 3EQM) based on its affiliation with breast cancer. In this study, computer-aided drug design molecular docking indicated that ziprasidone interacts with good binding affinities and best binding free energies with aromatase-targeted protein at its well-defined active sites. Greater negative values indicate the stronger binding, and our compound ziprasidone binds more strongly to aromatase receptor^32,33^. Therefore, with the help of advanced in-silico tools, it was predicted that ziprasidone could work as an anticancer drug agent. Identified compound ziprasidone is preeminent considered for the structure of naphthylpiperazine^34^. It also effectively inhibited Glutamic-Oxaloacetic Transaminase-1 (GOT1) in a non-competitive mode and can induce glutamine metabolism disorder and redox state imbalance of PDAC cells by specifically targeting GOT1, thereby it inhibits the cell growth, invasion, prevents the migration, and induces apoptosis^35^.It helps the patients afflicted with lifelong disease^36^.

The Aromatase Substrate undergoes rapid photobleaching in aqueous solutions^37^. In our experience, the linear phase begins in the kinetic mode for 60 min. To the best of our knowledge, our results can be explained the inhibitory activity of the produce compound (ziprasidone) against human aromatase enzyme.

The prediction of the anticancer drug potential of ziprasidone through in-silico approaches was further validated using in-vitro studies. For the in-vitro study, MCF-7, MDA MB 231 cell lines were selected because of their similarities with breast cancer of human origin^38^. To check the cytotoxic effect of ziprasidone on these cells, the tetrazolium-based MTT assay was performed^30^. MTT is a reliable and sensitive colourimetric technique, generally used for measuring in vitro cytotoxic effects of drugs on cancer cell lines and assessing the viability and proliferation of cancer cells^30^. Different doses of ziprasidone were selected to determine the most effective dose (ED) at a minimum concentration. Hence, we used concentrations ranging from 1, 0.5, 0.25, 0.125, and 0.0625 mM for 24 in-vitro studies. Cytotoxic effects of the compound ziprasidone observed using MTT assay on MCF-7, MDA MB 231, and T47D cells indicate dose and time-dependent activity. Our outcomes based on in-vitro studies indicate that treatment with ziprasidone causes the death of cancerous cells and that it could act as a cytotoxic agent in a concentration and time-dependent manner.

The ability of a drug to interact with DNA is a significant feature for it act as an anticancer agent. Such a compound targets the DNA molecule and interferes with the cell cycle, leading to cell death. Indeed, DNA interaction alters the cell’s fate by inhibition of replication and/or alteration of transcription. Our findings confirmed the arrest of the cell cycle, ultimately resulting in intrinsic and less likely extrinsic apoptosis. To study the anti-apoptosis effect induced by ziprasidone on the MCF-7, MDA-MB-231 and T47D culture cells, investigations were carried out through incubation of cells with AnnexinV-(FITC) and propidium. The results confirmed apoptosis in treated cells and they matched well with the morphological changes of the treated cells under the microscope. On the whole, our findings suggest that compound ziprasidone is as a potential anticancer agent.

In the present study, the compound, CHEMBL708, had better binding affinities based on the docking score. Toxicity and ADMET properties were predicted using computational calculations. The finding of this study suggests that this compound could be a potent inhibitor for the aromatase target. The efficiency of the selected compound will be further confirmed based on wet-lab experiments. The computational pipelines mentioned in this study would be beneficial in predicting possible potent inhibitory activities for novel drug candidates against the aromatase enzyme for use in breast cancer treatment. The in vitro enzymatic assays results suggest ziprasidone as a promising aromatase inhibitor. The cytotoxic activity of ziprasidone confirms its possible use as a chemotherapeutic agent. This compound was tested for its capability to induce cell cycle arrest and apoptosis in MCF-7, MDA MB 231 and T47D breast cancer cells. In conclusion, ziprasidone, through screening by in-silico molecular docking tools and in-vitro anticancer evaluation studies, emerged as a potential lead anticancer candidate for breast cancer.

## Methods

Our study is comprised of a regress in-silico analysis and in-vitro analysis. The in-silico analysis includes data collection, preparation and validation, molecular docking, MM\GBSA, molecular dynamics simulation and ADMET analysis. The in-vitro analyses are cell culture and drug treatment, cell proliferation assay, cell cycle analysis, and Annexin V-FITC/PI staining assay for apoptosis. A graphical abstract represents an understanding of the methods used. Further, the detailed methods are as follows-

### In silico studies

#### Protein structure prediction and validation

Numerous experimental structures were discovered in the protein data bank (PDB). The best one chemical structure that matched with aromatase enzyme was chosen from the RCSB PDB, freely available at www.rcsb.org^39^, based on the different parameters such as resolution, organism(s), methods (x-ray crystallographic structure, EM structure), mutation and other features. A three-dimensional structure of aromatase target protein (PDB ID 3EQM) defined as a target was selected, which contains Cytochrome P450 19A1 with X-ray diffraction 2.9 Å resolution and 503 amino acid residues. The structure was optimized, and energy minimization was performed by default constraint of 0.3 A of Root Mean Standard Deviation (RMSD) with OPLS force field using Macro model, Schrodinger suite, LLC, New York, NY, 2015. Hydrogen atoms were added to the targeted protein to retain the tautomeric states and stabilize the ionization of amino acid residues, and the Prime module was used to fill the missing residues, removed the ligand (HEM) and solvents and minimize the protein while using the OPLS force field^40^. The PROCHECK Ramachandran Plot Server was applied to analyze the geometry of the amino acids in the target protein, the conformation of the angles of the amino acid residues, and the interactions between the atoms^41,42^ and the prepared protein is shown in Fig. 1.

### Database preparation for bioactive conformation

Database preparation is crucial for finding the lead compound in the screening process. Researchers have constructed many libraries from large-scale bioactivity database ChEMBL (https://www.ebi.ac.uk/chembl/). These libraries constitute drug-like compounds and are analogous to a dictionary of small molecules with over 2 million compounds entries. The LigPrep (Schrodinger suite 2015) module was used for ligand preparation to assign the protonation states at biologically relevant pH. We prepared a library of 8506 compounds from the ChEMBL version-26 database, representing a significant source of chemical and biological information such as kind of binding, functional group and cellular activity, and ADMET data^22,43^. The library was downloaded in SDF format.

#### Active site prediction and receptor grid generation

Information on the binding sites of the amino acids is required for further molecular docking studies^44^. The protein’s active site was determined using Schrodinger’s Sitemap Programme^45^. The specific interaction of aromatase protein with five top ranking surface pockets was identified as a suitable binding site responsible for its drug-like compound cavity. The site with a Site score close to one was chosen for grid generation. The grid was generated using Glide v6.6, Schrodinger 2015. 3.1. We have taken grid box size with x range 22.67, y range 22.67, z range 22.67, and the lig x range 10, lig y range 10, and lig z range 10 for the optimization of the grid box to make the ligand dock in space.

#### Molecular docking

Docking experiments were conducted on Glide XP (extra precision) docking mode v6.6 module^45–47^ and the molecular mechanics\Generalized Born Surface Area (MM\GBSA)^48,49^ for the interaction of suitable complex of receptor-ligand structure was calculated via post-docking analysis called Prime in v3.6 Schrodinger Suite 2020. The ligand docking protocol examined the compound’s crucial phase of binding free energies^50–52^. It determines whether a ligand will bind or separate from the protein surface and return to its unbound state^51,53^. The docked complex was performed by evaluating the hydrogen bond interaction, hydrophobic interaction, pi–pi interaction, and pi-cation interaction in Schrodinger software.

Additionally, the docked complex was refined to calculate ΔG from MM-GBSA analysis^33^. The “MMGBSA *∆G* Bind” calculation [*dG* (1)] was done by the following equation:

$$dG\left( 1 \right) \, = \, E\_complex \, \left( {minimized} \right) \, - \, \left( {E\_ligand \, \left( {minimized} \right) \, + \, E\_receptor \, \left( {minimized} \right)} \right),$$

where the formula of MMGBSA designates molecular mechanics energies combined with the generalized Born and surface area continuum solvation; *dG* bind denotes computed free energy of ligand and receptor; *E_complex* is the MM/GBSA energy of the minimized complex, *E_receptor* represents MM/GBSA energy of protein (unbound, minimized) without ligand and *E_ligand* denotes the MM/GBSA energy of the ligand after removing it from the complex. We obtained reliable compounds based on docking score, MM-GBSA, and Qikprop module. The results are followed by (Tables 1 and 2). Further, molecular dynamic simulation was performed on docked protein–ligand complex.

#### ADMET investigation

ADME/Tox studies continue to drive the success of use of biological functions as targets for drug candidates. There are many reasons to estimate that 50% of drug candidates fail to win approval due to lack of efficiency and that up to 40% of drug candidates have failed in the past due to toxicity^54,55^. The analytical software QikProp module from Schrodinger suite 2020 was used for predicting the Absorption, Distribution, Metabolism and Excretion (ADME) properties which provide some important information related to the drugs/molecules. ADME properties were calculated for all molecules using QikProp 3.4 modules to identify promising ones that follow the bioavailability characteristics and ADMET profiling^27,55^. The criteria for ADME properties include SASA, FOSA, and FISA whose acceptable ranges were 300–1000, 0–750, and 7–330, respectively^56^. The total solvent-accessible volume range was from 500 to 2000; QPlogKhsa favourable range: from 1.5  to  1.5; molecular weight (mol MW): less than 500; Hydrogen bond donor and acceptor range: 0.0–6.0 and 2.0–20.0; QPlogHERG: acceptable range less than < − 5; QPPMDCK normal range: nm per sec, greater than 500; QPlogPC16 which used for projected the hexadecane/gas partition coefficient, recommended range: 4.0–18.0; octanol/water partition coefficient (QPlogPo/w), acceptable range: 3.069–3.905; QPlogPoct acceptable range: 8.0–35.0 (octanol/gas); QPlogKp normal range: − 8 to − 10; QPlogPw (water/gas) favourable range: 4.0–45.0; QPlogBB and QPPCaCo normal range: − 3 − 1.2 and (> 500)^28,57,58^.

#### Molecular dynamic simulation

Molecular dynamics is routinely applied to investigate dynamic properties and processes in structural biochemistry, pharmaceutical chemistry, molecular biology, enzymology, biophysics, and biotechnology. It is an invaluable tool extensively used to study the structure–function correlation in proteins^59^. It comprehensively considers several dynamic biomolecular structures’ characteristics, recognition, and function^50,60,61^. The molecular dynamics trajectory represents the computer simulation method for molecular systems, which provides the atomic coordinates at a specific period, single-point energies, and velocities^61^. Several algorithms exist for running MD simulations under different criteria^50,54^. MD simulation was performed using the Desmond *v3*.*6* package from Schrodinger, where we illustrated high density at the centre of a 10 × 10 × 10 Å orthorhombic box with the periodic frontier condition in NPT ensemble class in a buffer medium^62,63^. This module helps determine the RMSD value, Protein–Ligand torsion, protein–ligand interaction, validation, and optimization. Simulation time was set up to 100 ns with the recording of trajectories at each 100 ps interval and an orthorhombic box with a TIP3P water model to specify the shape. The system was further neutralized by adding the 5Cl- ion system charge. Temperature and pressure on the Kelvin scale were constant at 300 K in the equilibration period and 1.01325 bar, respectively^50^. The obtained trajectories were then analyzed using the Simulation Interaction Diagram (SID) tool and extensively analyzed the deviations, fluctuations and intermolecular interactions.

### Biological evaluation (in-vitro studies)

Based on the *in-silico* studies, we identified the lead compound as ziprasidone which is commercially available. This compound was selected for our in-vitro experimental studies.

#### Cell culture and drug treatment

Human breast (MCF-7, MDA-MB-231 and T47D) cancer cells were procured from National Centre for Cell Sciences (NCCS), Pune, India. The cells were maintained in Dulbecco’s Modified Eagle’s medium (HiMedia, Mumbai) with 10% FBS (Gibco, U.S.A.), penicillin 100 U/mL, streptomycin 100 mg/mL and amphotericin B 250 ng/mL at 37 °C in a humidified chamber consisting 5% CO_2_ and 95% air. Cells were then incubated with standard trypsinization (Trypsin: 0.25%) at70% confluency and sub-cultured in a 1/4 ratio for routine maintenance and experimentation. Compound ziprasidone was prepared in DMSO and exposed to the cells for 24h and 48h at a final volume of 0.1% DMSO.

#### Cell proliferation assay

The inhibitory effect of selected drugs was measured by MTT assay. Different breast cancer cell lines such as MCF-7, MDA-MB-231 and T47D were grown overnight in 96 cell culture flat bottom plates at a density of 8 × 10^3^ cells/well and treated with selected drugs from 0.62 to 2 mM for 24h and 48h. MTT (5mg/ml) was added with incomplete media at the end of incubation period for 3-4h. Subsequently, the medium was removed, dimethyl sulfoxide (100 µL/well) was added, and incubated for 5min at 37 °C under shaking conditions. Absorbance values at 570 nm were recorded using an ELISA plate reader, and the IC_50_ values were calculated from the dose–response curves.

#### Cell cycle analysis

Cell cycle progression was evaluated using Flowcytometry. MCF-7, MBA MD 231, and T47D human breast cancer cells were incubated for 48 h with the compound ziprasidone at IC50 value obtained by MTT assay. After 48 h of treatment, cells were harvested, washed twice with PBS, and were fixed in ice-cold 70% ethanol overnight at 4 ℃. The next day, all samples were centrifuged at 3000 rpm for 4 min. The cells were counterstained with propidium iodide (5 mg/ml) followed by the addition of RNase A and acquired on a spectrum flow cytometer (Cytek Aurora, Cytek Biosciences, Fremont, CA, USA) and DNA content was measured under blue laser (488nm), and a complete spectrum was captured in all 14 channels (B1-B14). The maxima were attained at 615/20 nm filter (B6). Data analysis was done by FlowJo software Version 10.8.1 (BD Biosciences, Ashland, OR, USA).

#### Annexin V/PI staining assay for apoptosis

The human breast cancer cell lines (MCF-7, MDA MB 231, and T47D) were seeded in 6-well plates at 10 × 10^4^ cells per well and grown overnight. Then cells were treated for 48 h with various compounds at IC50 value obtained by cell viability assay. After 48 h, cells were trypsinized, washed with Phosphate-buffered saline (PBS), and stained with annexin V-fluorescein isothiocyanate (FITC) and propidium iodide (PI) from Alexa Fluor 488 annexin V apoptosis detection kit (Beckman Coulter) using manufacturer protocol, followed by acquisition on a spectrum flow cytometer (Cytek Aurora, Cytek Biosciences, Fremont, CA, USA). Data analysis was done by FlowJo software Version 10.8.1 (BD Biosciences, Ashland, OR, USA).

#### In vitro aromatase enzymatic assay

The ability of the test compound (ziprasidone) to inhibit aromatase enzyme was performed using a aromatase Inhibitor Screening Kit (Catalog #K984-100; Store at − 20 °C) which is a high-throughput fluorescence-based assay kit enables reliable, rapid, characterization of drugs and other small molecules for compound-aromatase interaction with human aromatase^37^. The experimental protocol was followed according to the user manual. The highly fluorescent metabolite detected in the visible range (Ex/Em = 488/527 nm) of the test sample with different concentrations in kinetic mode were measured using a multimode microplate reader (Biotek Synergy H.T.X., USA) at room temperature upto 60 min. Duplicate samples for each measurement was carried out and the data were analyzed using GraphPad. Two time points (T1 and T2) in the linear phase of the reaction progress plot were chosen to obtain the corresponding fluorescence values at those points (RFU1 and RFU2) and determine

$$\Delta {\text{F }} = \, \left( {{\text{RFU2}}{-}{\text{RFU1}}} \right){\text{ and }}\Delta {\text{T }} = \, \left( {{\text{T2}}{-}{\text{T1}}} \right).$$

Calculated the rate of change in fluorescence over time according to the equation below: $\frac{\mathrm{\Delta F}-\mathrm{\Delta FBC}}{\Delta T},$where the subtract the rate of the background control denotated by BC; background-corrected reaction rates denoted by R for each well. We also calculated the percent inhibition using the following equation:

$$\% {\text{ Relative Inhibition }} = R_{SC} - R_{TC} /R_{SC} \times \, 100\% .$$

### Supplementary Information

Supplementary Information.

## Data Availability

The datasets used and/or analyzed during the current study are available from the corresponding author upon reasonable request.

## References

[1] Smith IE, Dowsett M (2003). Aromatase inhibitors in breast cancer. N. Engl. J. Med..

[2] Kizhakkeppurath Kumaran A (2023). Proteoglycans in breast cancer, identification and characterization by LC-MS/MS assisted proteomics approach: A review. Proteom. Clin. Appl..

[3] Michels KB (2002). The contribution of the environment (especially diet) to breast cancer risk. Breast Cancer Res..

[4] Travis RC, Key TJ (2003). Oestrogen exposure and breast cancer risk. Breast Cancer Res. BCR.

[5] Sahu A, Raza K, Pradhan D, Jain AK, Verma S (2023). Cyclooxygenase-2 as a therapeutic target against human breast cancer: A comprehensive review. WIREs Mech. Dis..

[6] Appert-Collin A, Hubert P, Crémel G, Bennasroune A (2015). Role of ErbB receptors in cancer cell migration and invasion. Front. Pharmacol..

[7] Arora A, Scholar EM (2005). Role of tyrosine kinase inhibitors in cancer therapy. J. Pharmacol. Exp. Ther..

[8] Atalay G, Cardoso F, Awada A, Piccart MJ (2003). Novel therapeutic strategies targeting the epidermal growth factor receptor (EGFR) family and its downstream effectors in breast cancer. Ann. Oncol..

[9] Yadav MK, Singh DB, Tripathi T (2023). Clinical applications of protein-based therapeutics. Protein-Based Therapeutics.

[10] Sahu A (2023). In silico screening, synthesis, characterization and biological evaluation of novel anticancer agents as potential COX-2 inhibitors. DARU J. Pharm. Sci..

[11] Kumavath R (2016). Novel aromatase inhibitors selection using induced fit docking and extra precision methods: Potential clinical use in ER-alpha-positive breast cancer. Bioinformation.

[12] Shaheenah D, Fellow SGK, Buzdar AU, Offermanns S, Rosenthal W (2008). Aromatase. Encyclopedia of Molecular Pharmacology.

[13] Lephart ED (2015). Modulation of aromatase by phytoestrogens. Enzyme Res..

[14] Anthoni H (2012). The aromatase gene CYP19A1: Several genetic and functional lines of evidence supporting a role in reading, speech and language. Behav. Genet..

[15] Mori T (2018). Aromatase as a target for treating endometriosis. J. Obstet. Gynaecol. Res..

[16] Altundag K, Ibrahim NK (2006). Aromatase inhibitors in breast cancer: An overview. Oncologist.

[17] Czajka-Oraniec I, Simpson ER (2010). Aromatase research and its clinical significance. Endokrynol. Pol..

[18] Nelson LR, Bulun SE (2001). Estrogen production and action. J. Am. Acad. Dermatol..

[19] Chan HJ, Petrossian K, Chen S (2016). Structural and functional characterization of aromatase, estrogen receptor, and their genes in endocrine-responsive and – resistant breast cancer cells. J. Steroid Biochem. Mol. Biol..

[20] Santen RJ, Brodie H, Simpson ER, Siiteri PK, Brodie A (2009). History of aromatase: Saga of an important biological mediator and therapeutic target. Endocr. Rev..

[21] Cava C, Castiglioni I (2020). Integration of molecular docking and in vitro studies: A powerful approach for drug discovery in breast cancer. Appl. Sci..

[22] Davies M (2015). ChEMBL web services: Streamlining access to drug discovery data and utilities. Nucleic Acids Res..

[23] Paul SM (2010). How to improve R&D productivity: The pharmaceutical industry’s grand challenge. Nat. Rev. Drug Discov..

[24] Lipinski CA (2016). Rule of five in 2015 and beyond: Target and ligand structural limitations, ligand chemistry structure and drug discovery project decisions. Adv. Drug Deliv. Rev..

[25] Lipinski CA (2000). Drug-like properties and the causes of poor solubility and poor permeability. J. Pharmacol. Toxicol. Methods.

[26] Shin HK, Kang Y-M, No KT, Leszczynski J (2016). Predicting ADME properties of chemicals. Handbook of Computational Chemistry.

[27] Cheng F, Li W, Liu G, Tang Y (2013). In silico ADMET prediction: Recent advances, current challenges and future trends. Curr. Top. Med. Chem..

[28] Shahbazi S (2016). Drug targets for cardiovascular-safe anti-inflammatory: In silico rational drug studies. PLoS One.

[29] Thakkar SS, Thakor P, Doshi H, Ray A (2017). 1,2,4-Triazole and 1,3,4-oxadiazole analogues: Synthesis, MO studies, in silico molecular docking studies, antimalarial as DHFR inhibitor and antimicrobial activities. Bioorg. Med. Chem..

[30] Tolosa L, Donato MT, Gómez-Lechón MJ (2015). General cytotoxicity assessment by means of the MTT assay. Methods Mol. Biol..

[31] Rieger AM, Nelson KL, Konowalchuk JD, Barreda DR (2011). Modified annexin V/propidium iodide apoptosis assay for accurate assessment of cell death. J. Vis. Exp..

[32] Halgren TA (2009). Identifying and characterizing binding sites and assessing druggability. J. Chem. Inf. Model..

[33] Zhang X, Perez-Sanchez H, Lightstone FC (2017). A comprehensive docking and MM/GBSA rescoring study of ligand recognition upon binding antithrombin. Curr. Top. Med. Chem..

[34] Gardner DM (2013). Evidence review and clinical guidance for the use of ziprasidone in Canada. Ann. Gen. Psychiatry.

[35] Yang Y (2022). Ziprasidone suppresses pancreatic adenocarcinoma cell proliferation by targeting GOT1 to trigger glutamine metabolism reprogramming. J. Mol. Med. (Berl.).

[36] Lombardino JG, Lowe JA (2004). The role of the medicinal chemist in drug discovery—Then and now. Nat. Rev. Drug Discov..

[37] Çevik UA (2022). Design, synthesis, and molecular modeling studies of a novel benzimidazole as an aromatase inhibitor. ACS Omega.

[38] Razak NA (2019). Cytotoxicity of eupatorin in MCF-7 and MDA-MB-231 human breast cancer cells via cell cycle arrest, anti-angiogenesis and induction of apoptosis. Sci. Rep..

[39] Berman HM (2000). The protein data bank. Nucleic Acids Res..

[40] Sastry GM, Adzhigirey M, Day T, Annabhimoju R, Sherman W (2013). Protein and ligand preparation: Parameters, protocols, and influence on virtual screening enrichments. J. Comput. Aided Mol. Des..

[41] Laskowski RA, MacArthur MW, Moss DS, Thornton JM (1993). PROCHECK: A program to check the stereochemical quality of protein structures. J. Appl. Cryst..

[42] Sahu A, Patra PK, Yadav MK, Varma M (2017). Identification and characterization of ErbB4 kinase inhibitors for effective breast cancer therapy. J. Recept. Signal Transduct. Res..

[43] Gaulton A (2012). ChEMBL: A large-scale bioactivity database for drug discovery. Nucleic Acids Res..

[44] Ghosh D, Griswold J, Erman M, Pangborn W (2010). X-ray structure of human aromatase reveals an androgen-specific active site. J. Steroid Biochem. Mol. Biol..

[45] Friesner RA (2004). Glide: A new approach for rapid, accurate docking and scoring. 1. Method and assessment of docking accuracy. J. Med. Chem..

[46] Ferreira LG, Dos Santos RN, Oliva G, Andricopulo AD (2015). Molecular docking and structure-based drug design strategies. Molecules.

[47] Friesner RA (2006). Extra precision glide: Docking and scoring incorporating a model of hydrophobic enclosure for protein-ligand complexes. J. Med. Chem..

[48] Genheden S, Ryde U (2015). The MM/PBSA and MM/GBSA methods to estimate ligand-binding affinities. Expert Opin. Drug Discov..

[49] Wang E (2019). End-point binding free energy calculation with MM/PBSA and MM/GBSA: Strategies and applications in drug design. Chem. Rev..

[50] Alonso H, Bliznyuk AA, Gready JE (2006). Combining docking and molecular dynamic simulations in drug design. Med. Res. Rev..

[51] Torres PHM, Sodero ACR, Jofily P, Silva-Jr FP (2019). Key topics in molecular docking for drug design. Int. J. Mol. Sci..

[52] Sahu A (2023). Computational screening for finding new potent COX-2 inhibitors as anticancer agents. Lett. Drug Des. Discov..

[53] Elokely KM, Doerksen RJ (2013). Docking challenge: Protein sampling and molecular docking performance. J. Chem. Inf. Model..

[54] Sahu A, Pradhan D, Raza K, Qazi S, Jain AK, Verma S (2020). In silico library design, screening and MD simulation of COX-2 inhibitors for anticancer activity. BICOB (EPiC Ser. Comput.).

[55] Norinder U, Bergström CAS (2006). Prediction of ADMET properties. ChemMedChem.

[56] Dasari T (2017). Design of novel lead molecules against RhoG protein as cancer target—A computational study. J. Biomol. Struct. Dyn..

[57] Ntie-Kang F (2013). An in silico evaluation of the ADMET profile of the StreptomeDB database. Springerplus.

[58] Egan WJ, Lauri G (2002). Prediction of intestinal permeability. Adv. Drug Deliv. Rev..

[59] Rana M (2021). Pyrazoline analogs as potential anticancer agents and their apoptosis, molecular docking, MD simulation, DNA binding and antioxidant studies. Bioorg. Chem..

[60] Karplus M, McCammon JA (2002). Molecular dynamics simulations of biomolecules. Nat. Struct. Biol..

[61] Adcock SA, McCammon JA (2006). Molecular dynamics: Survey of methods for simulating the activity of proteins. Chem. Rev..

[62] Ahmad S, Singh V, Gautam HK, Raza K (2023). Multisampling-based docking reveals Imidazolidinyl urea as a multitargeted inhibitor for lung cancer: An optimization followed multi-simulation and in-vitro study. J. Biomol. Struct. Dyn..

[63] Ahmad S, Raza K (2023). Identification of 5-nitroindazole as a multitargeted inhibitor for CDK and transferase kinase in lung cancer: A multisampling algorithm-based structural study. Mol. Divers..

