# Supplementary material for: In-silico and in-vitro study reveals ziprasidone as a potential aromatase inhibitor against breast carcinoma
Source: Sci Rep. 2023 Oct 2;13:16545. doi: 10.1038/s41598-023-43789-1 (PMC10545834; doi:10.1038/s41598-023-43789-1)
Supplement: Supplementary file 1 — Supplementary Information. [file 41598_2023_43789_MOESM1_ESM.docx]

***In-silico* and *in-vitro* study reveals Ziprasidone as a potential aromatase inhibitor against breast carcinoma**

**Ankita Sahu^1^, Shaban Ahmad^2^, Khalid Imtiyaz^3^, Ajeeshkumar Kizhakkeppurath Kumaran^1^, Mojahidul Islam^4^, Khalid Raza^2*^, Murugesh Easwaran^5^,** **Asha Kurukkan Kunnath^6^, Moshahid A. Rizvi^3^, Saurabh Verma^1#^**

**^1^** Tumour Biology Lab, ICMR-National Institute of Pathology, New Delhi-110029, India.

**^2^** Department of Computer Science, Jamia Millia Islamia, New Delhi-110025, India.

**^3^** Department of Bioscience, Jamia Millia Islamia, New Delhi-110025, India

**^4^** Molecular and Cellular Medicine, Institute of Liver and Biliary Sciences, Delhi-110070, India.

**^5^** Nutritional Improvement of Crops, Plant Molecular Biology Division, International Centre for Genetic Engineering and Biotechnology, New Delhi-110067, India.

^6^ICAR-Central Institute of Fisheries Technology, CIFT Junction, Matsyapuri PO, Cochin, 682029, India

**Co-Authors**

Ankita Sahu: ankitasahumbt@gmail.com (ORCID ID: 0000-0001-6679-3486)

Shaban Ahmad: shaban184343@st.jmi.ac.in (ORCID ID: 0000-0001-9832-2830)

Khalid Imtiyaz: khaliddar123@gmail.com (ORCID ID: 0000-0003-2854-5857)

Ajeeshkumar Kizhakkeppurath Kumaran: [ajeeshaksa@gmail.com](mailto:ajeeshaksa@gmail.com) ([ORCID ID: 0000-0003-4460-8969)](https://orcid.org/0000-0003-4460-8969)

Mojahidul Islam: islammojahidul4@gmail.com (ORCID ID: 0000-0001-7219-9686)

Khalid Raza: kraza@jmi.ac.in (ORCID ID: 0000-0002-3646-6828)

Murugesh Eswaran: murugeshphdsch@gmail.com (ORCID ID: 0000-0001-7628-0772)

Asha Kurukkan Kunnath: [asha.santhosh5@gmail.com](mailto:asha.santhosh5@gmail.com) (ORCID ID: 0000-0003-3688-3901)

Moshahid A. Rizvi: mrizvi@jmi.ac.in (ORCID ID: 0000-0002-4449-7819)

**^#^Corresponding Author**

**Dr Saurabh Verma**

Tumour Biology, ICMR-National Institute of Pathology, New Delhi, India-110029

**Contact:** svarmasv1@rediffmail.com/saurabhverma.nip@gov.in

ORCID ID: 0000-0003-1489-1871

***Co-Corresponding Author**

**Dr Khalid Raza**

Department of Computer Science, Jamia Millia Islamia -110025

**Contact:** Kraza@jmi.ac.in

ORCID ID: 0000-0002-3646-6828

***Supplementary Table 1 (ST1).*** *Showing the docking score and other computations of CHEMBL compounds against 3EQM.*

| Molecular Docking results of CHEMBL compounds against 3EQM | | | | | | |
| --- | --- | --- | --- | --- | --- | --- |
| CHEMBL ID | **Docking Score** | **Glide ligand efficiency sa** | **Glide ligand efficiency ln** | **Glide evdw** | **Glide ecoul** | **Rank (Docking)** |
| CHEMBL708 | -10.507 | -1.14 | -2.425 | -41.493 | -5.788 | 1 |
| CHEMBL598797 | -10.019 | -0.994 | -2.243 | -47.931 | -11.753 | 2 |
| CHEMBL1201387 | -9.603 | -1.017 | -2.199 | -38.926 | -3.768 | 3 |
| CHEMBL572878 | -9.263 | -0.9 | -2.06 | -43.018 | -8.232 | 4 |
| CHEMBL572878 | -9.263 | -0.9 | -2.06 | -43.018 | -8.232 | 5 |
| CHEMBL2106689 | -8.871 | -0.845 | -1.96 | -53.235 | -5.233 | 6 |
| CHEMBL34431 | -8.825 | -1.032 | -2.092 | -38.644 | -5.247 | 7 |
| CHEMBL495727 | -8.754 | -0.949 | -2.021 | -50.024 | -9.83 | 8 |
| CHEMBL495727 | -8.754 | -0.949 | -2.021 | -50.024 | -9.83 | 9 |
| CHEMBL708 | -8.637 | -0.937 | -1.994 | -45.34 | -5.054 | 10 |
| CHEMBL3301606 | -8.533 | -0.904 | -1.954 | -51.451 | -4.643 | 11 |
| CHEMBL223228 | -8.197 | -1.077 | -2.027 | -31.683 | -2.487 | 12 |
| CHEMBL3301606 | -8.068 | -0.855 | -1.847 | -51.113 | -4.883 | 13 |
| CHEMBL495727 | -8.001 | -0.868 | -1.847 | -44.173 | -11.729 | 14 |
| CHEMBL495727 | -8.001 | -0.868 | -1.847 | -44.173 | -11.729 | 15 |
| CHEMBL1521495 | -7.894 | -1.037 | -1.952 | -34.574 | -3.18 | 16 |
| CHEMBL138093 | -7.836 | -1.063 | -1.961 | -34.456 | -4.538 | 17 |

**Molecular Docking of Native Ligand of 3EQM for comparative studies-**

**Methods:** The same grid was used to dock the native ligand of PDBID: 3EQM and evaluated the results with same parameters.

**Results:**

The native ligand of 3EQM has produced only one hydrogen bond between MET374 and O atoms which is a smaller number of bonds and it also has produced less docking score along with the other computations (ST2 and SF1). Therefore, the Ziprasidone (CHEMBL708) has comparatively better score and inhibition capacity.

*Supplementary Table 2 (****ST2).*** *Showing the docking score (Kcal/mol) along with other computations of native ligand of 3EQM.*

| PDB | Docking Score | MM\GBSA | Prime Hbond | Prime vdW | mol MW | Ligand efficiency sa | Ligand efficiency In |
| --- | --- | --- | --- | --- | --- | --- | --- |
| 3EQM | -6.78 | -52.67 | -262.14 | -2277.83 | 286.413 | -0.891 | -1.676 |


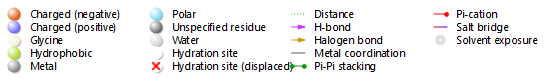

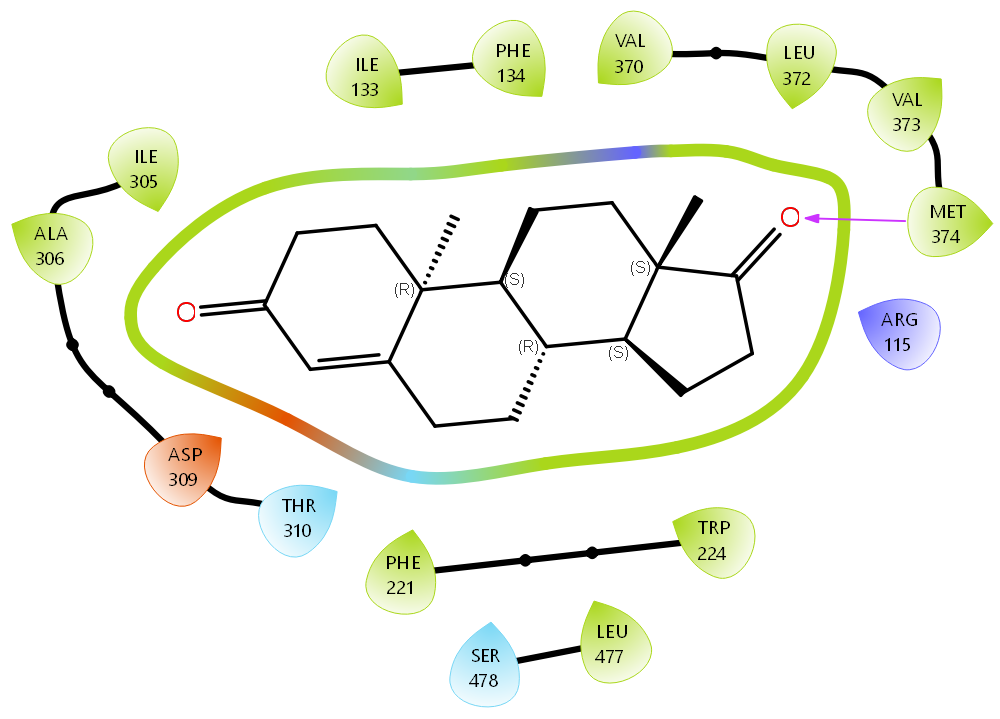


***Supplementary Figure 1 (SF1).*** *Showing the 2D interaction map of native ligand docked with 3EQM.*
